# High-Resolution Mapping of Tumor and Peritumoral Glutamate and Glutamine in Gliomas Using 7-T MRSI

**DOI:** 10.1148/rycan.240494

**Published:** 2025-09-12

**Authors:** Gilbert Hangel, Philipp Lazen, Cornelius Cadrien, Stefanie Chambers, Julia Furtner, Lukas Hingerl, Bernhard Strasser, Barbara Kiesel, Mario Mischkulnig, Matthias Preusser, Thomas Roetzer-Pejrimovsky, Adelheid Wöhrer, Wolfgang Bogner, Karl Rössler, Siegfried Trattnig, Georg Widhalm

**Affiliations:** ^1^High-field MR Center, Department of Biomedical Imaging and Image-guided Therapy, Medical University of Vienna, Vienna, Austria; ^2^Department of Neurosurgery, Medical University of Vienna, Währinger Gürtel 18-20, A-1090 Vienna, Austria; ^3^Christian Doppler Laboratory for MR Imaging Biomarkers, Vienna, Austria; ^4^Division of Neuroradiology and Musculoskeletal Radiology, Department of Biomedical Imaging and Image-guided Therapy, Medical University of Vienna, Vienna, Austria; ^5^Research Center for Medical Image Analysis and Artificial Intelligence (MIAAI), Faculty of Medicine and Dentistry, Danube Private University, Krems, Austria; ^6^Division of Oncology, Department of Internal Medicine I, Medical University of Vienna, Vienna, Austria; ^7^Division of Neuropathology and Neurochemistry, Department of Neurology, Medical University of Vienna, Vienna, Austria; ^8^Comprehensive Center for Clinical Neurosciences & Mental Health, Medical University of Vienna, Vienna, Austria; ^9^Institute for Pathology, Neuropathology, and Molecular Pathology, Medical University of Innsbruck, Innsbruck, Austria

**Keywords:** Glioma, 7 T, MR Spectroscopic Imaging, MRSI, Infiltration, Iisocitrate Dehydrogenase, *IDH*

## Abstract

**Purpose:**

To evaluate glutamate (Glu) and glutamine (Gln) concentrations in patients with glioma using 7-T MR spectroscopic imaging, identify significant differences in metabolic ratios between tumor and peritumoral regions, and assess associations of Glu and Gln with tumor-associated epilepsy and other tumor characteristics.

**Materials and Methods:**

This retrospective study included data from patients with gliomas who underwent 7-T MR spectroscopic imaging in a single university hospital between September 2018 and April 2021. Median values for nine metabolic ratios were calculated within the visible tumor and peritumoral shell, and Dice similarity coefficients were used to assess the spatial overlap of elevated metabolic regions between these compartments. Statistical significance between regions of interest and between glioma attributes (eg, isocitrate dehydrogenase status) was assessed.

**Results:**

Thirty-six patients (median age, 52 years [IQR, 23 years]; 22 male, 14 female) were included in the study. The Glu to total creatine (Glu/tCr) median was significantly higher in the peritumoral volume of interest (median, 1.13) compared with the tumor (median, 0.92; *P* = .00015) and normal-appearing white matter (NAWM; median, 0.87; *P* < .00011), while the Gln/tCr median was highest in the tumor (median, 0.77; peritumoral: median, 0.44; *P* < .00011; NAWM: median, 0.33; *P* < .00011). Glu to total choline was higher in the peritumoral region as well (median, 3.44; tumoral: median, 2.23; *P* < .00011; NAWM: median, 2.06; *P* < .00011). Peritumoral Dice similarity coefficients for Glu/tCr and Gln/tCr hotspots were comparable (0.51 to 0.53). Specific metabolic ratios were significantly different between isocitrate dehydrogenase mutant and wild-type gliomas (eg, tumoral Glu/total *N*-acetylaspartate [tNAA], *P* = .0054), oligodendroglioma and astrocytoma (eg, tumoral Gln/tNAA, *P* = .0033), and oligodendroglioma and glioblastoma (eg, tumoral Glu/tNAA, *P* = .0034).

**Conclusion:**

The 7-T MR spectroscopic imaging revealed increased Glu and Gln metabolic ratios within the peritumoral region compared with NAWM of patients with glioma distinct from intratumoral changes.

**Keywords:** Glioma, 7 T, MR Spectroscopic Imaging, MRSI, Infiltration, Iisocitrate Dehydrogenase, *IDH*

*Supplemental material is available for this article.*

© The Author(s) 2025. Published by the Radiological Society of North America under a CC BY 4.0 license.

## Graphical abstract

**Figure:**
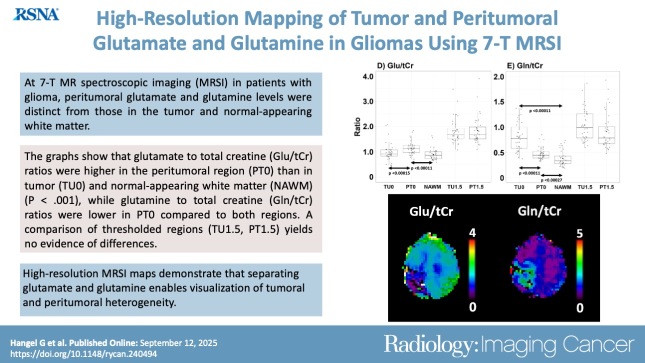

**Summary**

The 7-T MR spectroscopic imaging revealed increased glutamate and glutamine metabolic ratios within the peritumoral region compared with normal-appearing white matter in patients with glioma distinct from intratumoral changes, suggesting its potential clinical application for imaging glioma infiltration.

**Key Points**

■ At 7-T MR spectroscopic imaging in patients with glioma, peritumoral glutamate and glutamine levels were distinct from those in the tumor and normal-appearing white matter.
■ The median metabolic ratio of glutamate to creatine in the peritumoral region (median, 1.13) was higher than in the tumor region (median, 0.92; *P* = .00015) and the normal-appearing white matter region (median, 0.87; *P* < .00011), while glutamine-to-creatine ratios in the tumor region (median, 0.77) were higher than in the peritumoral region (median, 0.44; *P* < .00011) and the normal-appearing white matter region (median, 0.33; *P* < .00011).
■ Specific metabolic ratios were significantly different between isocitrate dehydrogenase mutant and wild-type gliomas (eg, tumoral glutamate to creatine, *P* = .0054), oligodendroglioma and astrocytoma (eg, tumoral glutamine to *N*-acetylaspartate, *P* = .0033), and oligodendroglioma and glioblastoma (eg, tumoral glutamate to *N*-acetylaspartate, *P* = .0034).

## Introduction

Diffuse gliomas, brain tumors originating from glial cells, account for the majority of malignant brain tumors (1). Their infiltrative growth into the peritumoral brain parenchyma limits treatment options, such as surgical resection and radiation therapy, contributing to high morbidity (eg, epilepsy [2] and mortality [3]). In clinical practice, structural MRI is used preoperatively to delineate the tumor infiltration zone and guide maximal safe resection. However, infiltrating glioma cells extend beyond regions visibly abnormal on T1-weighted, T2-weighted, and fluid-attenuated inversion recovery MR images. This infiltration into MRI-normal-appearing tissue leads to tumor recurrence.

As understanding of molecular and metabolic processes of gliomas has advanced, glioma classification has increasingly incorporated molecular markers. The 2021 World Health Organization (WHO) classification (4) recognized classes of gliomas as “astrocytoma, IDH-mutant,” “oligodendroglioma, IDH-mutant, and 1p/19q-codeleted,” or “glioblastoma, IDH-wildtype.” Isocitrate dehydrogenase (*IDH*) mutations lead to the accumulation of the oncometabolite 2-hydroxyglutarate, which is both a subject of ongoing research and a potential target for emerging clinical interventions. However, 2-hydroxyglutarate is not the only citric acid cycle-related metabolite relevant to gliomas.

Glutamate (Glu), a major excitatory neurotransmitter, is released in excessive and toxic amounts by infiltrating glioma cells, damaging surrounding healthy tissue and inducing necrosis (5–7). Glioblastoma cells can also migrate through peritumoral regions (5,6) and form synaptic connections with neurons, promoting tumor growth (8). This Glu activity (9) appears to be linked to α-amino-3-hydroxy-5-methyl-4-isoxazole propionic acid receptors and calcium signaling pathways in tumor and neuronal cells (5). Similar changes in Glu and calcium homeostasis have also been reported in astrocytomas (10). α-Ketoglutarate, the substrate for 2-hydroxyglutarate synthesis in *IDH* mutant tumors, can be produced from Glu (11). In *IDH* wild-type tumors, however, α-ketoglutarate–sourced Glu can be exchanged for extracellular cysteine. This process contributes to toxic Glu accumulation and enhances synthesis of the antioxidant glutathione (11).

Glutamine (Gln), synthesized from Glu and ammonia in astrocytes, serves as a precursor of Glu and γ-aminobutyric acid in neurons (12). Glioma cells exhibit increased dependence on Gln for metabolism, biosynthesis, and signaling (13). As a Glu precursor, Gln is also a precursor of α-ketoglutarate (14) and therefore 2-hydroxyglutarate (11). Direct deamidation of Gln by glioma cells releases not only cytotoxic Glu but also ammonia, which can cause cerebral edema and inhibit astrocytic Glu uptake, thereby promoting tumor growth (15). Notably, glioblastoma Gln levels have been reported to be higher in male individuals than in female individuals (16). Figure S1 provides a graphical summary of these metabolic connections.

Beyond its metabolic and tumor-promoting roles, Glu also contributes to the epileptogenicity of gliomas. Seizures are the most common symptom of gliomas at diagnosis (2,17), and release of Glu by infiltrating glioma cells plays a central role in tumor-associated epilepsy (TAE) (18). In human tumor and peritumoral tissue samples, increased Glu concentrations and Glu transporter expression were positively correlated with occurrence of preoperative seizures (19).

Given the critical role of Glu and Gln in glioma biology, imaging of their in vivo concentrations holds promise for both diagnostic and research applications. While both metabolites can be detected with MR spectroscopy (MRS), their spectral overlap makes them difficult to differentiate, requiring the combined signal to be reported as Glx (Glu plus Gln) (20). This limitation, along with inherent limitations of MRS in signal-to-noise ratio, scan times, and resolution, poses challenges for research and clinical use (21). Despite these limitations, studies have investigated Glu and Gln changes in gliomas. One study using 3-T MRS imaging (MRSI) reported reduced ratios of Glu to total creatine (Glu/tCr) and increased Gln/tCr compared with normal-appearing white matter (NAWM) (22). Another study using 3-T single-voxel spectroscopy reported differences in intratumoral Glx concentrations across WHO 2017 grade 2, 3, and 4 gliomas (20) and the association of higher Glu/tCr with shorter progression-free survival and overall survival (23). Additional 3-T single-voxel spectroscopy studies in glioblastoma found that an intratumoral Glu/tCr cutoff of more than 1.81 predicted postsurgical seizures with an area under the receiver operating characteristic curve of 0.82 (24) and that peritumoral Glu/tCr was significantly increased in patients with TAE (25). Overall, peritumoral Glu and Gln in patients with glioma remain understudied, largely due to the limited spatial resolution and coverage of conventional 3-T MRS methods.

A new generation of MRSI combines improved signal-to-noise ratio and spectral separation at 7 T with fast spatial-spectral encoding and free induction decay acquisition (26,27). This technique enables acquisition of a wider array of three-dimensional neurochemical maps within 15 minutes at 3.4-mm isotropic resolution. With its ability to separately image oncometabolites at high resolution, 7-T MRSI has revealed intratumoral heterogeneity for Glu, Gln, total choline (tCho), and other metabolites such as glycine (28,29). Importantly, this method allows simultaneous mapping of tumor and peritumoral regions and separates Gln and Glu, enabling investigation of Glu and Gln changes relevant to glioma infiltration and epileptogenesis.

The purpose of this study was to evaluate Glu and Gln concentrations in patients with glioma using 7-T MRSI, identify significant differences in metabolic ratios between tumor and peritumoral regions, and assess associations of Glu and Gln with TAE and other tumor characteristics.

## Materials and Methods

### Patients and Clinical Data

This retrospective study included 42 patients with glioma who underwent preoperative 7-T MRSI scans in a single university hospital between September 2018 and April 2021. The study was approved by the institutional review board, and all patients provided written informed consent. The study sample was either partially (28–30) or fully (31) analyzed in previous publications, as detailed in Table S1. These previous studies differed from the current one, as they did not conduct a quantitative analysis of the peritumoral region using MRSI; instead, the studies involved qualitative analysis of only high-grade gliomas using MRSI (28), comparison of MRSI to PET (29), comparison of MRSI to MR fingerprinting (30), and determination of *IDH* mutation status with MRSI-based machine learning (31). For the 7-T MRSI scans, inclusion criteria were a Karnofsky performance status of 70 or more, and exclusion criteria were 7-T MRI contraindications (eg, pregnancy, claustrophobia, ferromagnetic implants, nonferromagnetic metal head implants > 12 mm). Additional criteria for inclusion in this study were histologically confirmed gliomas (2021 WHO classification [4], Table S1) and sufficient 7-T data quality, as detailed in Cadrien et al (31).

We further collected clinical preoperative MRI data, consisting of noncontrast and contrast-enhanced (gadoteridol, 0.1 mmol/kg) T1-weighted imaging, T2-weighted imaging, and T2-weighted fluid-attenuated inversion recovery imaging.

### 7-T MRSI Measurement and Processing

We conducted all MRSI scans on a 7-T scanner (Magnetom or Magnetom.plus after an upgrade; Siemens Healthineers) with a 1Tx32Rx head coil (Nova Medical). Before MRSI, we acquired T1-weighted MRI (MP2RAGE) and fluid-attenuated inversion recovery scans.

Our MRSI sequence (26,28,31) used free-induction-decay acquisition and concentric ring trajectories to acquire a 64 × 64 × 39 matrix (field of view of 220 mm × 220 mm × 133 mm, resolution of 3.4 mm × 3.4 mm × 3.4 mm) in 15 minutes. Further parameters were repetition time of 450 msec, acquisition delay of 1.3 msec, 39° excitation flip angle, 345-msec readout duration with 2778-Hz spectral bandwidth, 7-T–optimized WET (water suppression enhanced through T1 effects) water suppression (32), and field of view placement maximizing tumor coverage in the superior parts of the brain, which are less affected by B_0_-inhomogeneities.

We processed the MRSI raw data offline in a custom pipeline including iMUSICAL coil combination (26), k-space regridding (26,33), and coil-wise L2-regularization (34) to remove lipid signals. The resulting spectra were quantified per voxel using LCModel (35) (basis set: tCr, cysteine, γ-aminobutyric acid, Gln, Glu, glycine, glutathione, tCho, myo-inositol, serine, taurine, *N*-acetylaspartate [NAA], NAA-Glu, macromolecular baseline [36]; 1.8–4.1 ppm range; separation performance of Glu and Gln were discussed previously [28]). The resulting intensities (in institutional units) were aggregated into three-dimensional metabolic maps. As MRSI concentration estimation within the tumor was not possible without a knowledge of intratumoral voxel T_1_ and water concentrations, we calculated ratio maps. For our evaluation, we selected the ratios of Glu/total NAA [tNAA] (NAA plus *N*-acetylaspartylglutamate), Gln/tNAA, Glu/tCr, Gln/tCr, Glx/tNAA, Glx/tCr, Glu/tCho, and Gln/Glu to investigate tumoral and peritumoral activity of Glu and Gln. As a reference, we added the clinical standard ratio of Cho/tNAA.

For spectral quality evaluation, we calculated voxel-wise, full-width-at-half-maximum, and signal-to-noise ratio of tCr at 3.02 ppm as well as Cramér-Rao lower bounds of all metabolites (37). More details, such as voxel exclusion criteria, are summarized according to the MRSinMRS standard in Table S2 (38) and described in previous publications (26,28,31).

### Coregistration and Volumes of Interest Definition

We coregistered 3-T and 7-T images using the Medical Imaging Interaction Toolkit before segmentation of tumor volumes (TU, defined as binary “visible tumor including edema yes/no” to avoid creating too many small volumes of interest [VOIs] and subgroups by separating contrast enhancement and necrosis) by a neuroradiologist (J.F., 17 years of relevant experience) based on the 3-T protocols (28) in ITK-SNAP (version 3.2; University of Pennsylvania). We then resampled TU to the MRSI resolution. We also defined a peritumoral VOI (PT) made up of six iterations of dilation (corresponding to 2 cm) of TU.

Using 7-T T1-weighted images for a gray matter–white matter segmentation, we defined a NAWM reference VOI by removing TU and PT from white matter and eroding the result once. We also removed gray matter voxels from PT to allow a white matter–centric comparison without higher-Glu gray matter effects.

For the subsequent evaluation using a Python script, we used five VOIs per metabolite ratio: NAWM as above including ratios 0 < ratio_voxel_ < 10 (to avoid outliers); TU0 and PT0 using the same range to evaluate overall VOI trends; and TU1.5 and PT1.5, which used 1.5 × (median NAWM ratio per metabolite) < ratio_voxel_ < 10 to look at metabolic hotspots.

### Statistical Analysis and Evaluation

In the selected ratios, we conducted four analyses using R (version 4.4.2; R Foundation) and Python (version 3.8.10; Python Software Foundation) for statistical differences of median ratios between VOIs, categorical differences per ratio and VOI, Sørensen–Dice similarity coefficients (DSCs) between TU1.5 and PT1.5, and correlations between categories and ratios. We applied a Bonferroni correction of nine to two statistical test threshold levels that determined significance (.05 → .0055; .001 → .00011).

### Differences between VOI Medians

For all five VOIs and all nine ratios, we calculated median ratios. We applied Mann-Whitney *U* tests for the median differences between the VOI pairs TU1.5 and PT1.5, TU0 and PT0, TU0 and NAWM, and PT0 and NAWM. We excluded other combinations, as they would be significantly different due to different thresholding. In a subanalysis, we repeated these tests for the male and female patients independently.

### Categorical Differences

For all four tumor VOIs and all selected ratios, we evaluated independent two-sided *t* tests for the categorical pairs “male/female,” “TAE Yes/no,” “*IDH* mutation/*IDH* wildtype,” “low grade/high grade,” “astrocytoma/oligodendroglioma,” “astrocytoma/glioblastoma,” and “oligodendroglioma/glioblastoma.”

### Similarity Coefficients

We calculated patient-wise DSCs of the TU1.5 and PT1.5 VOIs from the original TU and PT segmentations as well as the DSCs of Glu/tNAA to Gln/tNAA, Glu/tCr to Gln/tCr, and Glu/tNAA to Cho/tNAA using the following formula:

$$DSC=\frac{2\times \left|N_{1}\cap N_{2}\right|}{\left|N_{1}\right|+\left|N_{2}\right|}$$

with |*N*_1_| and |*N*_2_| as voxel amounts. A DSC of 1 describes a complete overlap of two VOIs, while a DSC of 0 signifies none. We then calculated median DSCs for the study sample.

## Results

### Patient Characteristics

**Figure 1:**
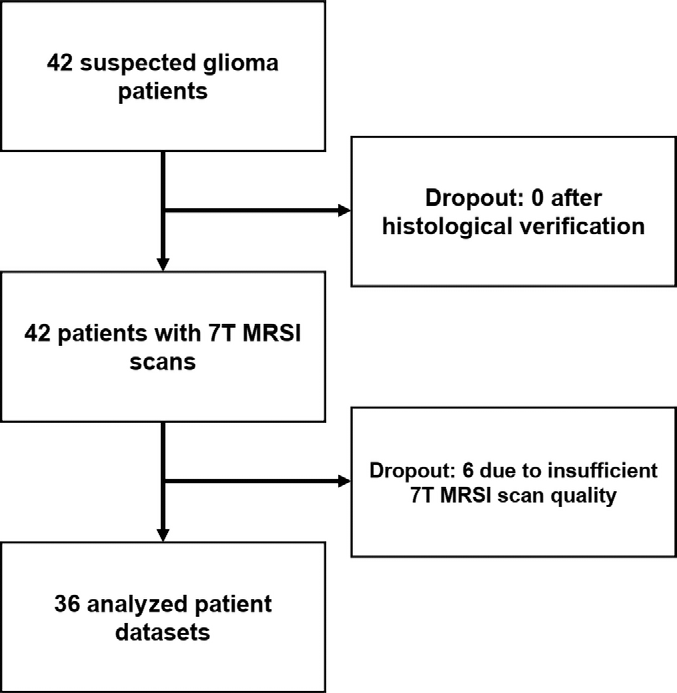
Patient inclusion flowchart. MRSI = MR spectroscopic imaging.

After exclusion of six patients due to poor 7-T MRSI scan quality, the study included 36 patients with glioma (median age, 52 years [IQR, 23 years]; 22 male, 14 female) (Fig 1). The study sample included 16 patients with *IDH* wild-type glioblastoma, 14 with *IDH *mutant astrocytoma, and six with *IDH *mutant 1p/19q codeleted oligodendroglioma. In total, 28 patients had high-grade (ie, WHO grade 3 or 4) and eight had low-grade (ie, WHO grade 2) gliomas. Detailed patient characteristics are presented in Table S1.

### Metabolic Ratios

**Figure 2:**
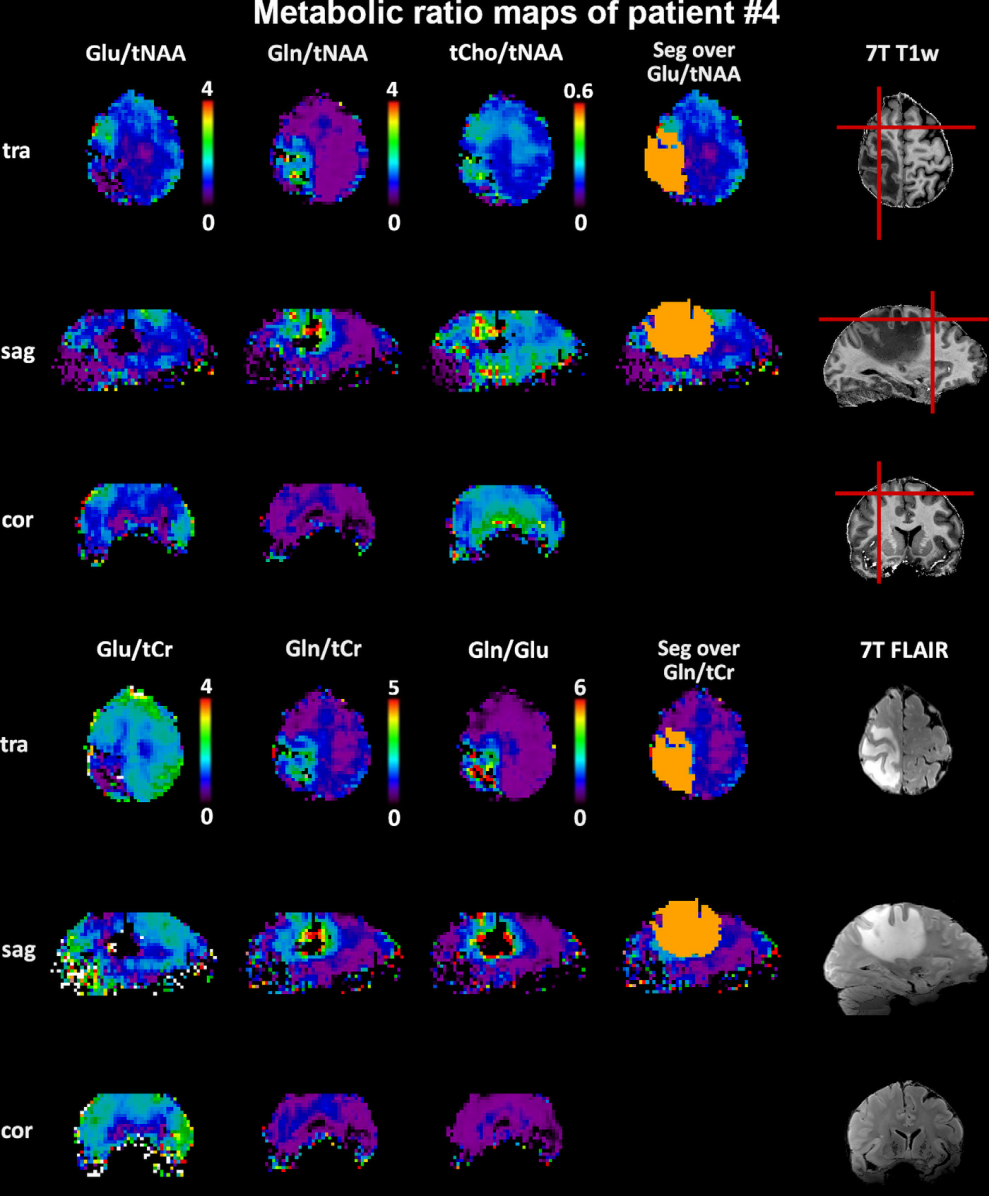
Exemplary transversal (tra), sagittal (sag), and coronal (cor) metabolic ratio maps along segmentation and 7-T noncontrast T1-weighted (T1w) and fluid-attenuated inversion recovery (FLAIR) images (in a 47-year-old female patient with glioblastoma, isocitrate dehydrogenase wild type) that display tumoral and peritumoral glutamate (Glu) and glutamine (Gln) distributions. Of note is a frontal peritumoral Glu/total *N*-acetylaspartate (tNAA) hotspot that has no correspondence on the Gln ratio maps. These maps also show the general power of Gln-based maps to discern tumor metabolism versus the healthy-appearing brain with a contrast of over a magnitude in this case. Red lines indicate the location of the MR spectroscopic imaging (MRSI) sections. Seg = tumor segmentation, tCho = total choline, tCr = total creatine.

Figure 2 shows an example of Glu/tNAA, Gln/tNAA, and tCho/tNAA, highlighting peritumoral regions in a 47-year-old female patient with glioblastoma. Figure S1 shows example spectra for TU, PT, and NAWM. Table S3–S6 contain the patient-wise medians, DSCs, and thresholds used in this analysis.

### Differences between VOI Medians

Median TU ratios were higher than PT ratios for both thresholds, except for Glu/tCr in TU0 (median, 0.92) versus PT0 (median, 1.13) versus NAWM (median, 0.87) and Glu/tCho for both (TU0 [median, 2.23] vs PT0 [median, 3.44] vs NAWM [median, 2.06]; TU1.5 [median, 4.34] vs PT1.5 [median, 4.51]) (Table 1). In comparison, Gln/tCr medians were 0.77 in TU0, 0.44 in PT0, and 0.33 in NAWM. All TU/PT VOIs were higher than in NAWM for the 0 threshold except tCho/tNAA in PT0 versus NAWM (and obviously so for the 1.5 threshold). These distributions are also plotted in Figure 2, visualizing the differences between ratios to tCr and tNAA. The differences between Glu/tNAA and Glu/tCr showed the impact of the chosen reference metabolite, as the tumoral decrease of tNAA masked the apparent increase of Glu in PT0. Combining Glu/tCr and Gln/tCr into Glx/tCr masked their diverging distributions. High similarity between the Gln/Glu and tCho/tNAA distributions was visible as well.

**Table 1:** Median Values of all Evaluated Ratios over the TU1.5, PT1.5, TU0, PT0, and NAWM VOIs

Metabolite Median	TU1.5	PT1.5	TU0	PT0	NAWM
Glu/tNAA	0.98 (1.08–0.83)	0.88 (0.98–0.80)	0.72 (0.79–0.54)	0.61 (0.68–0.55)	0.42 (0.47–0.37)
Gln/tNAA	0.70 (0.84–0.56)	0.40 (0.54–0.36)	0.61 (0.73–0.45)	0.26 (0.29–0.22)	0.18 (0.20–0.12)
Glu/tCr	1.68 (1.93–1.52)	1.67 (1.98–1.51)	0.92 (1.08–0.83)	1.13 (1.25–0.97)	0.87 (1.01–0.74)
Gln/tCr	0.99 (1.26–0.78)	0.78 (1.01–0.67)	0.77 (0.99–0.58)	0.44 (0.51–0.39)	0.33 (0.43–0.27)
Glx/tNAA	1.46 (1.73–1.28)	1.18 (1.31–1.14)	1.26 (1.47–1.03)	0.85 (0.93–0.76)	0.56 (0.63–0.50)
Glx/tCr	2.49 (2.77–2.07)	2.38 (2.80–2.04)	1.60 (1.98–1.35)	1.56 (1.69–1.40)	1.20 (1.38–1.01)
Glu/tCho	4.34 (4.96–3.80)	4.51 (5.49–4.21)	2.23 (3.09–1.78)	3.44 (3.94–2.83)	2.06 (2.48–1.81)
Gln/Glu	1.18 (1.27–0.98)	0.86 (1.09–0.75)	0.81 (1.01–0.61)	0.41 (0.46–0.35)	0.39 (0.49–0.32)
tCho/tNAA	0.49 (0.55–0.41)	0.39 (0.44–0.33)	0.29 (0.41–0.21)	0.18 (0.20–0.16)	0.20 (0.22–0.17)

Note.—IQRs are reported in parentheses. Glutamate to total creatine (Glu/tCr) and Glu/total choline (tCho) are higher in peritumoral segmentation without ratio threshold (PT0) than in tumor segmentation without ratio threshold (TU0) and normal-appearing white matter (NAWM). Gln = glutamine, Glx = glutamate plus glutamine, PT1.5 = peritumoral segmentation with 1.5 × normal-appearing white matter threshold, tNAA = total *N*-acetylaspartate, TU1.5 = tumor segmentation with 1.5 × normal-appearing white matter threshold, VOI = volume of interest.

**Figure 3:**
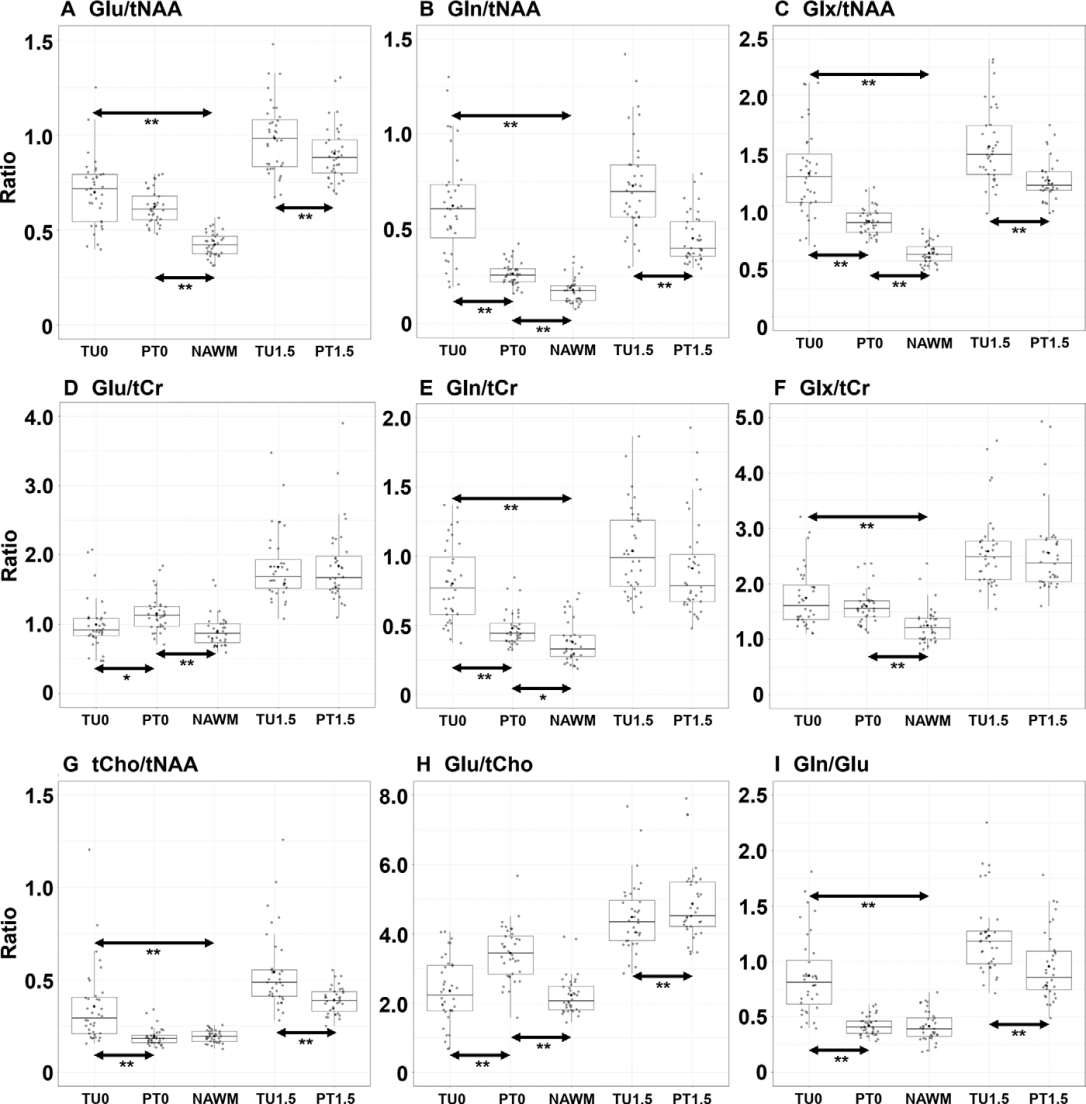
**(A–I)** Box plots of all evaluated ratios over the volumes of interest that include statistically significant differences. All ratios could discern at least two compartments. * indicates *P* < .0055 and ** indicates *P* < .00011. Gln = glutamine, Glu = glutamate, Glx = glutamate plus glutamine, NAWM = normal-appearing white matter, PT0 = peritumoral segmentation without ratio threshold, PT1.5 = peritumoral segmentation with 1.5 times normal-appearing white matter threshold, tCho = total choline, tCr = total creatine, tNAA = total *N*-acetylaspartate, TU0 = tumor segmentation without ratio threshold, TU1.5 = tumor segmentation with 1.5 times normal-appearing white matter threshold. Center line is median and box is 25th to 75th percentiles. Whiskers are 75th + 1.5 IQR or highest value on top and 25th − 1.5 IQR or lowest value on bottom. Dots represent the individual values.

The statistical significances of these differences (Table 2, Fig 3) elaborate on these findings. The reference ratio tCho/tNAA could distinguish TU0 from PT0 (*P* < .00011) and NAWM (*P* < .00011) but not PT0 from NAWM (*P* = .5). All ratios with Glu and Gln except Gln/Glu significantly differed between PT0 and NAWM (all *P* < .00011 except Gln/tCr with *P* = .00027) (Table 2). For TU0 and NAWM, all ratios except Glu/tCr and Glu/tCho were significantly different (*P* < .00011). All ratios, except Glx/tCr and Glu/tNAA, could significantly distinguish between PT0 and TU0 (all *P* < .00011 except Glu/tCr with *P* = .00015). The thresholded PT1.5 and TU1.5 VOIs had Glu/tNAA, Gln/tNAA, Glx/tNAA, Glu/tCho, Gln/Glu, and Cho/tNAA as significant ratios (all *P* < .00011). Overall, Gln ratios showed more significant differences than Glu ratios except for PT0 to NAWM. The subanalyses of male and female subgroups found male results to be only different in additional significance of Glu/tCr in TU1.5 versus PT1.5 (*P* = .0042) but found nine less significant changes in the female subsample (Table S7).

**Table 2:** Distribution of *P* Values for Median Ratios between Different VOIs

VOI	Glu/tNAA	Gln/tNAA	Glu/tCr	Gln/tCr	Glx/tNAA	Glx/tCr	Glu/tCho	Gln/Glu	Cho/tNAA
TU1.5 to PT1.5	<.00011	<.00011	.5	.0095	<.00011	.9	<.00011	<.00011	<.00011
TU0 to PT0	.0063	<.00011	.00015	<.00011	<.00011	.052	<.00011	<.00011	<.00011
TU0 to NAWM	<.00011	<.00011	.036	<.00011	<.00011	<.00011	.5	<.00011	<.00011
PT0 to NAWM	<.00011	<.00011	<.00011	.00027	<.00011	<.00011	<.00011	.6	.5

Note.—All thresholds were modified by a Bonferroni correction of nine to account for multiple testing (resulting in *P* < .00011 [.001 before correction] and *P* < .0055 [.05 before correction]). These results show that glutamate (Glu) and glutamine (Gln) ratios in most cases can discern between tumor, peritumoral region, and normal-appearing white matter (NAWM). Gln/total *N*-acetylaspartate (tNAA) and glutamate plus glutamine (Glx)/tNAA do so in all cases, while the reference total choline (tCho)/tNAA cannot discern between NAWM and peritumoral region. Cho = choline, PT0 = peritumoral segmentation without ratio threshold, PT1.5 = peritumoral segmentation with 1.5 × normal-appearing white matter threshold, tCr = total creatine, TU0 = tumor segmentation without ratio threshold, TU1.5 = tumor segmentation with 1.5 × normal-appearing white matter threshold, VOI = volume of interest.

### Categorical Differences

**Figure 4:**
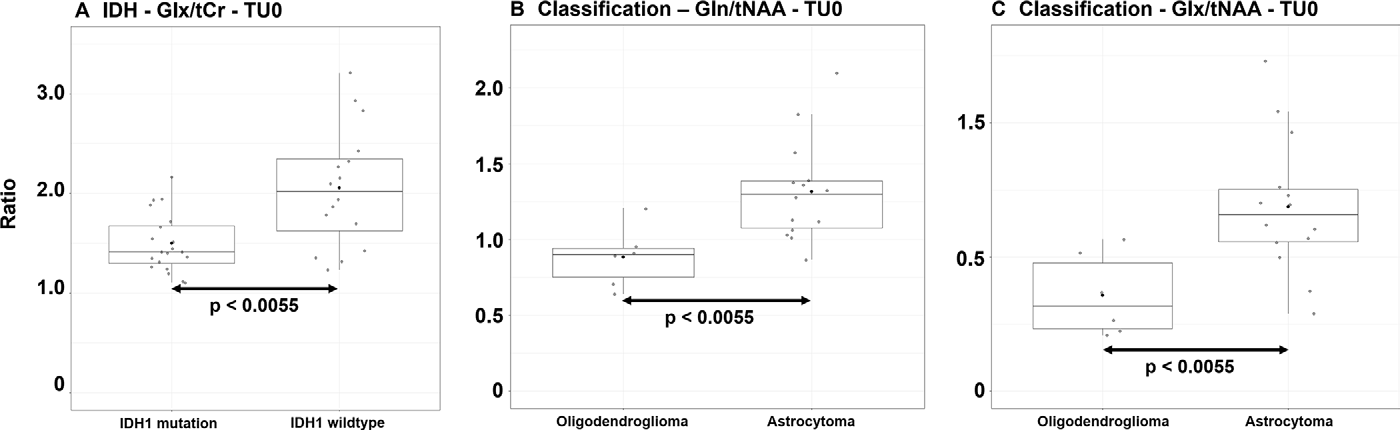
Box plots of three example ratios. **(A)** Glutamate plus glutamine to total creatine (Glx/tCr), **(B)** glutamine to total *N*-acetylaspartate (Gln/tNAA), and **(C)** Glx/tNAA, all in tumor segmentation without ratio threshold (TU0), for which we found significant categorical differences. *IDH* = isocitrate dehydrogenase. Center line is median and box is 25th to 75th percentiles. Whiskers are 75th + 1.5 IQR or highest value on top and 25th − 1.5 IQR or lowest value on bottom. Dots represent the individual values.

**Figure 5:**
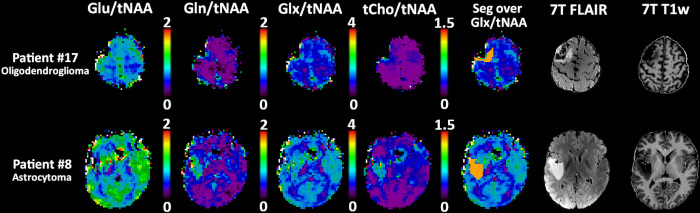
Comparison of transversal metabolic ratio maps along segmentation and 7-T noncontrast T1-weighted (T1w) and fluid-attenuated inversion recovery (FLAIR) images between a patient (no. 17, a 56-year-old male) with oligodendroglioma, isocitrate dehydrogenase mutant, and 1p/19q codeleted and a patient (no. 8, a 33-year-old male) with astrocytoma and isocitrate dehydrogenase mutant, where the median peritumoral glutamine to total *N*-acetylaspartate (Gln/tNAA) and glutamate plus glutamine (Glx)/tNAA ratios differed. Glu = glutamate, Seg = tumor segmentation, tCho = total choline.

We found significant differences for *IDH* mutation/*IDH* wildtype in TU0 Glu/tCr (*P* = .0054) and Glx/tCr (*P* = .0035). We found significant differences for astrocytoma/oligodendroglioma in TU0 Gln/tNAA (*P* = .0033) and Glx/tNAA (*P* = .0046) and in PT0 Gln/tNAA (*P* = .0023) and Glx/tNAA (*P* = .0015). We also found significant differences for oligodendroglioma/glioblastoma in TU1.5 Glx/tNAA (*P* = .0036), in TU0 Glu/tNAA (*P* = .0034) and Glx/tNAA (*P* = .0024), and in PT0 Gln/tNAA (*P* = .0024). Three of these distributions are plotted in Figure 4. Figure 5 gives an example of such differences for astrocytoma versus oligodendroglioma. For TAE Yes/no, low grade/high grade, astrocytoma/glioblastoma, and male/female, we found no significant differences.

### Similarity Coefficients

**Figure 6:**
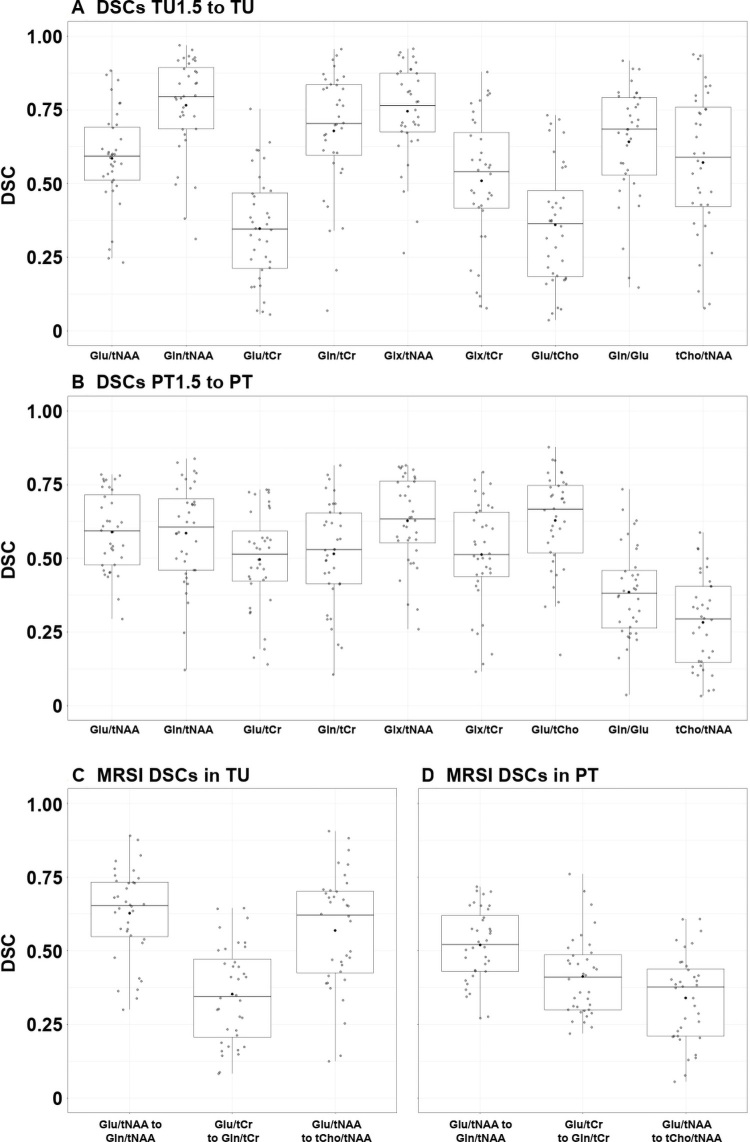
**(A–D)** Box plots of all calculated Dice similarity coefficients (DSCs). Cho = choline, Gln = glutamine, Glu = glutamate, Glx = glutamate plus glutamine, MRSI = MR spectroscopic imaging, PT = peritumoral volume of interest, PT1.5 = peritumoral volume of interest with 1.5 times normal-appearing white matter threshold, tCho = total choline, tCr = total creatine, tNAA = total *N*-acetylaspartate, TU = tumor volume of interest, TU1.5 = tumor volume of interest with 1.5 times normal-appearing white matter threshold. Center line is median and box is 25th to 75th percentiles. Whiskers are 75th + 1.5 IQR or highest value on top and 25th − 1.5 IQR or lowest value on bottom. Dots represent the individual values.

Of all resulting DSCs to the TU and PT segmentation VOIs (representing how much the thresholded hotspot filled them out, Table 3, Fig 6), TU Gln/tNAA had the highest DSC (0.79), even greater than tCho/tNAA (0.59), which is consistent with previous research (29). DSCs to TU were generally higher than those to PT (eg, Glx/tNAA with 0.76 to 0.63), except for Glu/tCr (0.34 to 0.51) and Glu/tCho (0.36 to 0.67). Glu ratio DSCs were lower than Gln DSCs but were generally higher in PT than in TU (eg, Glu/tCr with 0.51 to 0.34 compared with Gln/tCr with 0.53 to 0.70). For the DSCs between ratios, Glu/tNAA to Gln/tNAA was 0.65 in TU1.5 and 0.52 in PT1.5, and Glu/tCr to Gln/tCr was 0.34 in TU1.5 and 0.41 in PT1.5. This finding implies that Glu and Gln ratio hotspot overlap is driven by tNAA decreases.

**Table 3:** Median DSCs for the TU1.5 and PT1.5 Thresholds

Metabolite DSCs	TU1.5	PT1.5
Glu/tNAA to VOI	0.59 (0.69–0.51)	0.59 (0.71–0.48)
Gln/tNAA to VOI	0.79 (0.89–0.68)	0.60 (0.70–0.46)
Glu/tCr to VOI	0.34 (0.47–0.21)	0.51 (0.59–0.42)
Gln/tCr to VOI	0.70 (0.83–0.60)	0.53 (0.65–0.41)
Glx/tNAA to VOI	0.76 (0.88–0.68)	0.63 (0.76–0.55)
Glx/tCr to VOI	0.54 (0.67–0.42)	0.51 (0.66–0.44)
Glu/tCho to VOI	0.36 (0.48–0.18)	0.67 (0.75–0.52)
Gln/Glu to VOI	0.68 (0.79–0.53)	0.38 (0.46–0.26)
Cho/tNAA to VOI	0.59 (0.76–0.42)	0.29 (0.40–0.15)
Glu/tNAA to Gln/tNAA	0.65 (0.73–0.55)	0.52 (0.62–0.43)
Glu/tCr to Gln/tCr	0.34 (0.47–0.21)	0.41 (0.49–0.30)
Glu/tNAA to Cho/tNAA	0.62 (0.70–0.43)	0.38 (0.44–0.21)

Note.—IQRs are reported in parentheses. Glutamine to total *N*-acetylaspartate (Gln/tNAA) to the tumor segmentation volume of interest (VOI) scored the highest, while the reference ratio total choline (tCho)/tNAA to the peritumoral segmentation scored the lowest. Cho = choline, DSC = Dice similarity coefficient, Glu = glutamate, Glx = glutamate plus glutamine, PT1.5 = peritumoral segmentation with 1.5 × normal-appearing white matter threshold, tCr = total creatine, TU1.5 = tumor segmentation with 1.5 × normal-appearing white matter threshold.

## Discussion

Investigating the peritumoral distribution of Glu and Gln for the first time with high-resolution 7-T MRSI, we found statistically significant changes compared with NAWM in the peritumoral area beyond radiologically visible neoplasm and edema. Glu and Gln distributions show relevant differences between the compartments, while the standard MRS biomarker tCho/tNAA cannot discriminate PT0 and TU0, but our interest lies in where Glu and Gln maps diverge. Based on the literature, we expect this to be related to the interaction of infiltrating glioma cells with brain parenchyma leading to Glu buildup versus increased Gln metabolism in the visible tumor (5,6,9,10,12,13). The clinical implications of imaging these processes lie in an improved delineation of glioma progress and treatment.

In detail, comparing Glu/tNAA to Gln/tNAA showed that both were significantly different between PT0 and both TU0 and NAWM, but for Glu/tNAA, PT0 is closer to TU0 than to NAWM. Comparing Glu/tCr to Gln/tCr showed significantly higher values in PT0 over the other regions in Glu/tCr, while Gln/tCr decreased from TU0 to NAWM. This finding demonstrated the impact of the chosen reference for metabolic ratios. While Glu/tCr was higher in PT0, Glu/tNAA was higher in TU0, indicating that the tNAA decrease in the segmented tumor masked Glu distribution. Glu/tCho offers an interesting alternative to investigate peritumoral activity as we found it significantly different between PT0 and TU0 or NAWM, likely due to the contrast of infiltrative interaction with the brain parenchyma versus membrane activity in the segmented tumor. Gln/Glu, however, increased only in TU0 but not in PT0, which completes the picture that Glu and Gln increases in PT0 are of similar magnitude, but Gln increases in TU0 are much more pronounced while Glu relatively decreases there. This finding could be based on different Glu-Gln exchange rates in different microenvironments and the interaction of infiltrating glioma cells with the brain parenchyma (5,6,11–13).

That these findings generally replicate in the male subsample but not in the female one could hint at sex-based differences, but as that subsample is notably smaller (14 vs 22), we cannot rule out a lack of statistical power. Increased sample sizes, such as in multicentric studies, should shed more light on this aspect in the future.

The most interesting finding for the DSCs was that Glu/tCr and Glu/tCho were higher in PT1.5 than in TU1.5, the higher similarity score representing in this case that these ratio VOIs covered a higher percentage of the peritumoral volume than within the tumor. This finding again shows that separating Glu and Gln by 7-T MRSI resolves oncometabolic heterogeneities not only within the visible tumor volume. This insight could have a clinical utility for the optimization of resection margins and follow-up monitoring. Even with the limited accessibility of clinical 7-T systems, validated by more research, this could be a new use case for second-level scans after an initial diagnosis. Significant differences between oligodendrogliomas and both astrocytomas and glioblastomas, but not between the last two, point at greater metabolic complexities that require more exploration. A final insight is that the application of a hotspot threshold is less necessary than expected to detect differences. Yet a more thorough analysis of glioma compartments and larger subsamples could clarify findings of significance.

Our results agree with previous literature about the role of Glu and Gln in glioma metabolism (5,6,12,13). Compared with 3-T MRS studies, we confirmed increased tumoral Gln/tCr (22) but did not find decreased Glu/tCr compared with NAWM (22).

Compared with previous qualitative research (28), we could quantize observations about Glu and Gln increases in gliomas. Two other 7-T MRS studies (39,40) found decreased Glu/tCr and no significant changes for Gln/tCr when comparing tumor to the precuneus but found a similar tumoral increase in Gln/Glu as in our work (39) as well as increased Gln/tCr but decreased Glu/tCr between tumor and NAWM (40). In total, the state-of-the-art in MRS remains unclear due to different MRS methods, field strengths, and reference regions.

In summary, our results show that the 7-T MRSI enhanced spatial resolution and ability to separately map Glu and Gln improves imaging assessment of gliomas. Our findings of significant Glu and Gln changes in the peritumoral region compared with tumor and NAWM regions could be the starting point for clinical quantification of glioma infiltration and the investigation of glioma-induced epilepsy.

This study had limitations. Our analysis was mainly limited by sample size (an issue of nearly all glioma MRS studies), specifically in the female subsample, and it also lacked methodologic consensus on the definitions of tumor VOIs (such as edemas or infiltration), control regions, hotspots, and reference standards, limiting our statistical power (eg, for the relation of TAE to metabolism). Relying on metabolic ratios introduces unknowns and can hide or create distinct changes, such as NAA decreasing to below the detectability threshold.

In conclusion, this study showed that 7-T MRSI, due to higher resolution and Glu-Gln separation, could investigate peritumoral glioma regions and infiltration. In a pathology with abysmal outcomes and the inability of clinical standard MRI to define infiltration extent, we could add value to the clinical description and research into gliomas. For instance, we propose the scouting of points of interest for surgical sampling during resection or the monitoring of prospective new drugs such as *IDH* inhibitors (eg, vorasidenib) or Glu synthesis inhibitors (eg, troriluzole). Further ahead, Glu and Gln maps, together with other data such as perfusion maps, could be inputs for synthetic infiltration extent images. This spatial resolution would not be feasible with single-voxel spectroscopy or low-resolution MRSI. Methodologically, further developments to investigate the importance of Glu and Gln imaging could include quantitative MRSI, which would require proton-density scans as a reference but would allow for disentangling ratios. Continuing to increase our sample size and to better define VOIs (including sufficient datasets to analyze different grades with respect to contrast enhancement and necrosis and to compare edema to the peritumoral region) for analysis will clarify our findings. Meanwhile, Gln/Glu and Glu/tCho could be explored for their use in the definition of the properties of infiltrative gliomas.
